# Supplementary material for: Leveraging mobility data to analyze persistent SARS-CoV-2 mutations and inform targeted genomic surveillance
Source: eLife. 2025 Jan 15;13:RP94045. doi: 10.7554/eLife.94045 (PMC11735024; doi:10.7554/eLife.94045)
Supplement: Supplementary file 2. [file elife-94045-supp2.zip › supplementary_file_2supplementary_method.docx]

# Phylogenetic time tree construction

7,262 sequenced Alpha lineage genomes with less than 12,000 ambiguous bases were exported with the respective data from our local database. These were filtered to remove 609 non-Thuringian isolates, four duplicates, and 127 entries missing location information or isolation date. After filtering, the Thuringian dataset contained 6,522 isolates.

From the 1,091,655 public German genomes provided by the Robert Koch Institute, 146,347 Alpha lineage isolates were extracted. For these, more specific location data (country, state, district, city, latitude, longitude) were added based on the sending laboratory’s postal code. Subsequently, 9,600 Thuringian isolates were removed. The resulting 136,747 genomes were then analyzed with poreCov (version 1.7.2) to determine mutations/deletions and confirm the lineages.[^1^](https://www.zotero.org/google-docs/?A1Nxh8) The results were uploaded to a separate collection in our local database. poreCov identified a further 108 non-Alpha isolates, which were subsequently removed from the dataset. During the generation of a test phylogenetic time tree (see below), “Nextstrain” excluded 540 isolates due to quality issues.[^2^](https://www.zotero.org/google-docs/?wrZqqQ) After their removal, the final German Alpha dataset comprised 136,099 entries.

The prepared Thuringian and German Alpha lineage datasets were combined to build a phylogenetic time tree using the program “Nextstrain” (version 5.0.0).[^2^](https://www.zotero.org/google-docs/?kiYYXW) Following the program's instructions, two genomes of the original Wuhan lineage were added to the combined fasta-file, while their respective data were added to the combined data file (omitting this step would lead to a crash in the used Nextstrain version). It was ensured that the data file contained the necessary columns given in the manual. Further, in our version, the data file needed to contain at least 12 columns, or Nextstrain would have an error. The resulting fasta and metadata file for the nextstrain analysis are provided as Supplementary Files 3 and 4, respectively.

A custom “build.yaml”-file was used for the nextstrain analysis, which is provided as Supplementary File 5:

inputs:

- name: alpha-lin_phylotree

metadata: data/alpha-lin_phylotree_metadata.tsv

sequences: data/alpha-lin_phylotree.fasta

builds:

thuringia:

subsampling_scheme: custom-division

region: Europe

country: Germany

division: Thuringia

subsampling:

custom-division:

focal:

group_by: "year month"

seq_per_group: 10000000

query: --query "(country == '{country}') & (division == '{division}')"

related:

group_by: "country year month"

seq_per_group: 16000

exclude: "--exclude-where 'division={division}'"

priorities:

type: "proximity"

focus: "focal"

files:

auspice_config: "my_profiles/alpha-lin_analysis/my_auspice_config.json"

description: "my_profiles/alpha-lin_analysis/my_description.md"

The analysis was started with the command:

“nextstrain build . --cores 32 --profile my_profiles/path/to/build/dir”.

The resulting phylogenetic time tree comprises 64,131 German (non-Thuringian) and 6,298 Thuringian Alpha-lineage genomes. Additional 2,160 German (non-Thuringian) and 224 Thuringian genomes are included in the metadata but not visualized in the tree and, therefore, not considered for the following analysis. The resulting tree is shown in Figure 1-figure supplement 3. The subsampled genome-, tree-, and metadata file are provided as Supplementary Files 6, 7, and 8.

## References

[1 Brandt C, Krautwurst S, Spott R, *et al.* poreCov-An Easy to Use, Fast, and Robust Workflow for SARS-CoV-2 Genome Reconstruction via Nanopore Sequencing. *Front Genet* 2021; **12**: 711437.](https://www.zotero.org/google-docs/?9F4Uto)

[2 Hadfield J, Megill C, Bell SM, *et al.* Nextstrain: real-time tracking of pathogen evolution. *Bioinformatics* 2018; **34**: 4121–3.](https://www.zotero.org/google-docs/?9F4Uto)
